# Primary Amenorrhea Due to Anatomical Abnormalities of the Reproductive Tract: Molecular Insight

**DOI:** 10.3390/ijms222111495

**Published:** 2021-10-25

**Authors:** Karina Kapczuk, Witold Kędzia

**Affiliations:** Division of Gynecology, Department of Perinatology and Gynecology, Poznan University of Medical Sciences, 60-535 Poznan, Poland; witold.kedzia@poczta.fm

**Keywords:** primary amenorrhea, congenital absence of uterus and vagina, Müllerian aplasia, MRKH syndrome, DSD

## Abstract

Congenital anomalies of the female reproductive tract that present with primary amenorrhea involve Müllerian aplasia, also known as Mayer–Rokitansky–Küster–Hauser syndrome (MRKHS), and cervical and vaginal anomalies that completely obstruct the reproductive tract. Karyotype abnormalities do not exclude the diagnosis of MRKHS. Familial cases of Müllerian anomalies and associated malformations of the urinary and skeletal systems strongly suggest a complex genetic etiology, but so far, the molecular mechanism in the vast majority of cases remains unknown. Primary amenorrhea may also be the first presentation of complete androgen insensitivity syndrome, steroid 5α-reductase type 2 deficiency, 17β-hydroxysteroid dehydrogenase type 3 deficiency, and Leydig cells hypoplasia type 1; therefore, these disorders should be considered in the differential diagnosis of the congenital absence of the uterus and vagina. The molecular diagnosis in the majority of these cases can be established.

## 1. Introduction

Congenital malformations of the female genital organs that present with primary amenorrhea involve a congenital absence of the uterus and vagina (CAUV) and some obstructive anomalies of the reproductive tract. Surgical correction of complete uterine or vaginal obstruction results in the occurrence of menses. On the contrary, amenorrhea due to CAUV is irreversible; therefore, this complex malformation is considered the most severe anomaly of the female reproductive tract. Disorders of gonadal development that trigger gonadal dysgenesis and premature ovarian insufficiency as a cause of primary amenorrhea are not the subject of this review.

The most common cause of CAUV (class U5C4V4 of the European Society of Human Reproduction (ESHRE)/the European Society of Gynecological Endoscopy (ESGE) classification [1]) is Müllerian aplasia, also known as Mayer–Rokitansky–Küster–Hauser syndrome (MRKHS). Isolated Müllerian aplasia is classified as MRKHS type 1 (typical form). Müllerian aplasia associated with at least one concomitant congenital malformation is classified as MRKHS type 2 (atypical form).

Utero-vaginal aplasia was also reported as a cause of primary amenorrhea in patients diagnosed with other syndromes of multiple congenital anomalies. Considering the variety of extragenital malformations attributed to MRKHS type 2, in some cases, the phenotypic features of these patients might overlap. Cat-eye syndrome (CES) (OMIM 115470) is one such example. CES is characterized by ocular coloboma, periauricular tags, and anal atresia and results from the presence of extra copies of the pericentromeric 22q11 region [2]. On the other hand, duplications at the chromosomal region 22q11.21 also have been identified by array comparative genomic hybridization (CGH) in patients with MRKHS [3]. In both syndromes, renal and heart malformations are frequent. At least two patients with the association of CES and Müllerian aplasia have been reported [2]. Next, complete Müllerian agenesis (absent fallopian tubes and uterus) was found in two sisters with familial Beckwith–Wiedemann syndrome (BWS) caused by the CDKN1C gene mutation [4]. Uterus aplasia was also described in two syndromes that present with shortening of the upper limbs: the Al-Awadi-Raas-Rotschild syndrome (AARRS) (OMIM 276820) [5] and the thrombocytopenia-absent radius (TAR) syndrome (OMIM 274000) [6,7]. The *WNT7A* loss-of-function mutations cause AARRS, and its main clinical futures include short upper limbs with ulnar deficiency, oligodactyly, hypoplastic nails, and reduction defects of the lower limbs [5]. The key characteristics of TAR syndrome, caused by the deletion of 1q21.1 or mutation at the *RBM8A* gene, involve the absence of the radius, preservation of the thumbs, and thrombocytopenia [6,7]. On the other hand, in women with MRKHS, radial aplasia was reported as an associated finding [8,9]. Deletions affecting the TAR susceptibility locus and mutations of the *RBM8A* gene, both located in 1q21.1, were found in patients with MRKHS [10].

The spectrum of diseases that might affect phenotypic females with CAUV and therefore, warrant consideration in the differential diagnosis of MRKHS also involve the following: complete androgen insensitivity syndrome (CAIS), steroid 5α-reductase type 2 (SRD5A2) deficiency, 17β-hydroxysteroid dehydrogenase type 3 (HSD17B3) deficiency, and Leydig cells hypoplasia (LCH) (Table 1). These conditions belong to the spectrum of 46,XY disorders (differences) of sex development (46,XY DSD). In contrast to patients with MRKHS, patients with these disorders have 46,XY karyotype and gonads corresponding to testes. The female phenotype of these patients results from undervirilization, due to abnormal androgen action or synthesis.

## 2. Embryogenesis of the Female Genital Tract

In humans, the Müllerian (paramesonephric) ducts (MDs) and the Wolffian (mesonephric) ducts (WDs) are essential for the development of the female and male genital systems, respectively. Although the WDs do not contribute cells to the developing MDs, they are required for proper MDs development [11]. The MDs are of mesodermal origin, and their formation can be divided into three phases: specification, invagination, and elongation. During the first phase, Müllerian precursor cells appear within the coelomic epithelium covering the cranial pole of the mesonephros (the Müllerian surface epithelium, MSE), adjacent to the WD [12]. The initiation phase of MDs formations is regulated by a sequential action of BMP/PAX2 and FGF/LIM1 signaling [13]. During the second phase, the Müllerian duct–specified cells, PAX2/LIM1 positive, invaginate and proliferate in a caudal direction through the mesenchyme (the Müllerian duct mesenchyme, MDM) between the coelomic epithelium and WD [12]. Invagination requires WNT4, which is produced and released from the MDM cells [12]. During the third phase, the invaginating cells form the Müllerian duct epithelium (MDE), come into physical contact with the WD, and form the canalized tube that, through proliferation and migration of the MDE cells, grows caudally alongside the WD until it ultimately fuses with the urogenital sinus [12]. WNT4 and WNT9B, derived from the neighboring WD, are major factors required for MD elongation [14]. At the point where the MDs contact the endodermal urogenital sinus, called the Müllerian tubercule, the urogenital sinus epithelium proliferates and forms the sinovaginal bulbs that finally give rise to the lining epithelium of the vagina [15]. The Müllerian ducts fuse and form the midline uterovaginal canal [16]. In males, MD regression is driven by anti-Müllerian hormone (AMH), a member of the TGFbeta growth factor family secreted by Sertoli cells in the developing testes. AMH binds to its receptors type II (AMHR2) and I (AMHR1) in the MDM. In females, the MDs differentiation into fallopian tubes, uterus, cervix, and upper portion of the vagina is regulated by WNT7A and segmental (from cranial to caudal direction) expression of genes *Hoxa9*, *Hoxa10*, *Hoxa11*, and *Hoxa13* [14,17].

## 3. MRKH Syndrome

**Figure 1:**
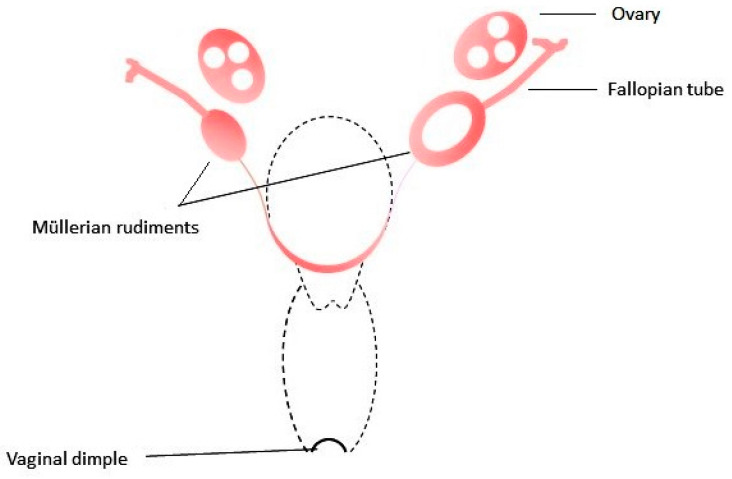
MRKH syndrome (utero-vaginal aplasia, class U5 C4 V4 of the ESHRE/ESGE classification). The dotted line is the contour of absent uterus and vagina.

MRKHS is characterized by uterovaginal aplasia or hypoplasia in an otherwise phenotypically normal female with a normal 46,XX karyotype (OMIM 277000). MRKHS is a rare disease that affects about 1:5000 newborn females (range 1:4000–10,000) [18]. Nevertheless, it is the second most common cause of primary amenorrhea. The phenotypic heterogeneity of patients with MRKHS is mainly determined by a spectrum of associated congenital extragenital anomalies and, to a lesser degree, by the severity of Müllerian aplasia within the range from the complete absence of vaginal opening, vagina, and uterus to the presence of blind-ending shallow vagina and rudimentary uterine horns with a functional endometrium (Figure 1). The proportion of patients with coexisting malformations, usually renal and skeletal defects, varies in different populations. In the largest published cohort of MRKHS women of Chinese origin, 70% (734/1055) of the women had type 1 MRKHS, and the remaining 30% (321/1055) had type 2 MRKHS [19]. In the largest European cohort of 346 MRKHS women from Germany, a proportion of patients with concomitant malformations was higher (41% had MRKHS type 2 and further 5.5% had MURCS (Müllerian aplasia, renal aplasia, cervicothoracic somite anomalies) association) [8]. The most striking is the difference in the proportion of patients with renal anomalies. Kidney malformations were diagnosed in nearly 10% of the Chinese MRKHS women [19], while in the European cohorts, 27% to 34% of MRKHS women had concomitant renal malformations [8,18]. In some cohorts of patients, skeletal anomalies, most commonly spinal and of upper limbs, preponderated over renal anomalies (22% affected patients in the mentioned Chinese cohort [19]; 32% versus 29% in the Polish cohort [9]).

Although the definition of MRKHS includes normal female karyotype 46,XX, chromosomal aberrations were reported in patients with MRKHS. Rall et al. [8] found abnormal karyotypes in 5 of 346 (1.4%) patients with MRKHS. The abnormalities involved the following: weak Turner mosaicism (45,X[3]/46,XX[37]), a subtelomeric deletion or unbalanced translocation of the chromosomal region 21q22.3 (46,XX,del(21) (q22.3)), Robertsonian translocation of chromosomes 14 and 15 (46,XX, Robertsonian translocation (14;15)), a pericentric inversion of chromosome 9 (46,XX,inv(9) (p11q13)) and a marker chromosome (47,XX,mar[13]/46XX[2]). We found abnormal karyotypes in 4 out of 125 (3.2%) patients with MRKHS, and the abnormalities involved the following: low-level Turner mosaicism (45,X[3]/46,XX[47]), a triple X (47,XXX), low-level mosaicism with presence of Y chromosome (46,XY[2]/46,XX[98]), inversion of chromosome 9 (46,XX, inv(9)(p12q21)) and low level mosaicism of 3 chromosomal rearrangements, including translocation (46,XX, t(1;14)(p11;q11)) and deletion (46,XX,del(5)(p11→pter) [9]. Chromosomal abnormalities, including 46,X/46,XX mosaicism and 47,XXX, were also identified in 2.8% of Dutch women with MRKHS [18]. The coexistence of MRKHS with Turner syndrome was also described [20].

Reports from different studies that searched genomic rearrangements by genome-wide array CGH have revealed chromosomal abnormalities in a larger number of MRKHS cases (about 10–15%) [3,21,22]. The identified recurrent chromosomal imbalances that are considered the regions harboring potentially causative genes for MRKHS typically are microdeletions or microduplications in the chromosomal regions 17q12 (with likely causative genes, *LHX1* and *HNF1B*), 16p11.2 (with likely causative gene, *TBX6*), 22q11, 1q21 (with likely causative gene, *RBM8A*) and Xp22, with the most frequent 1.2–1.9 Mb deletions at 17q12 [21,22]. Nevertheless, the pathogenic contribution of these chromosomal changes has not been completely elucidated, and the genotype–phenotype correlations remain unobvious.

The genetic base of MRKHS is largely unsolved, though many candidate genes have been suggested. The molecular determinants of MRKHS remain unknown in the majority of patients. Both phenotypic complexity of the patients and the results of genetic studies that address the etiology of MRKHS indicate that the genetic background of this syndrome is very complex, and the influence of epigenetic and environmental factors cannot be excluded [23]. The latter is mainly based on cases of monozygotic twins discordant for Müllerian aplasia. In the research that involved the largest reported cohort of MRKHS-discordant monozygotic twins, differences in copy number variants (CNVs) patterns between the affected and non-affected siblings were found only in one of the five pairs of twins [24]. The identified duplication of chromosome 14q11.2 was considered significant, as the region contained genes of matrix metalloproteinase 14 *(MMP14*) and low-density lipoprotein receptor-related protein 10 (*LRP10*) with known relevant roles during embryonic development of the female reproductive tract [24]. Nevertheless, despite the fact that familial cases of MRKHS have been reported [25], most cases are sporadic.

The *WNT4* gene mutation was the first one found to cause MRKHS. The first mutation found and proved causative of MRKHS concomitant with hyperandrogenism without clinical signs of virilization (OMIM 158330) was the Q226G missense mutation of the WNT4 protein, due to a heterozygous substitution of guanine for adenine in exon 5 of the *WNT4* gene [26]. However, it is worth mentioning that androgen excess is quite common in MRKHS women but is rarely associated with the *WNT4* gene mutations. In a study that analyzed the hormonal status of 215 MRKHS patients, hyperandrogenemia was found in 52% of the subjects, but no relevant mutations or single nucleotide polymorphisms (SNPs) were detected in the *WNT4* gene [27]. So far, three causative *WNT4* mutations (p.R83C, p.L12P, p.A233T) were identified in adolescents with MRKHS and hyperandrogenism [28,29].

Exome sequencing (ES), which was performed in one of the largest cohorts of patients with MRKHS, involving 442 Chinese individuals and 150 European and American individuals, in 4.4% (26/592) of the patients identified 12 loss-of-function variants in 7 candidate genes: *PAX8*, *BMP4*, *BMP7*, *TBX6*, *HOXA10*, *EMX2*, and *WNT9*. *PAX8* represented the most significant disease-associated gene, underlying the etiology of MRKHS in 1.2% (7/592) of the patients; in four patients, the variants were paternally inherited, which is consistent with an autosomal dominant mode of inheritance with the sex-dependent phenotypic expression of the disease trait [30]. *PAX8* is also critical for the development of the thyroid gland; microdeletion of 2q12.1q14.1, involving *PAX8*, was described in a patient with congenital hypothyroidism, due to thyroid gland hypoplasia [31]. Another study based on whole ES (WES), conducted in a group of 111 patients with MRKHS and focused on 72 candidate genes, revealed deleterious candidate variants in 10 genes: *WNT4*, *LAMC1*, *RARA*, *HOXA10*, *PAX2*, *WNT9B*, *TBX6*, *SHOX*, *MMP14*, and *LRP10*, nearly all of which were heterozygous [32]. The WES of diagnostic and candidate genes undertaken in eight women with MRKHS identified 16 genomic variants in 14 genes; the two affecting *LRP10* and *DOCK4* genes were considered potentially contributory but had no proof from functional studies [33]. Conversely, the deleterious variants of the *GEN1* gene were found and were demonstrated to cause Müllerian aplasia, acting as a single pathogenic variant or synergistically in the combinations with the deleterious variants of other genes (mainly *WNT9B* gene) [34]. Disease-causing mutations were also identified in the *LHX1*, also known as *LIM1* gene [35,36]. Lastly, causative variants in the *GREB1L* gene (growth regulation by estrogen in breast cancer 1 like) were identified in individuals with MRKHS, mainly type 2 with concomitant anomalies of kidney or urinary tract [37]. The other potential candidate genes associated with MRKHS are the *OXTR* and the *ESR1* genes [38]. The variants that potentially impair the oxytocin receptor and the estrogen receptor-1 function were identified in MRKHS patients, supporting the hypothesis that impaired estrogen receptor function could cease the development of the Müllerian ducts at the attachment of the caudal mesonephric ligament (later the round ligament) [38,39].

Rall et al. [40] compared the whole-genome expression and methylation patterns in the myometrium of uterine rudiments from patients with MRKHS and normal uterine tissue from healthy controls. The overexpression of ESR1 and progesterone receptor (PGR) and hypomethylation of CpG sites within *WT1* and *GATA4* were found in the rudimentary uterine tissue of MRKHS patients, leading to activation of the *AMH* gene during embryological development, and thus, are responsible for MD regression. The hypomethylation of specific CpG sites and corresponding overexpression of *HOXA9* and *HOXA5* was also found to prevent normal differentiation of the MDs. The authors pointed out that the involvement of endocrine disruptors (ED), especially with estrogen-like functions, might mimic this effect [40].

The prevalence of potentially pathogenic mutations in the MRKHS-susceptibility genes in the studied cohorts of MRKHS women was very low, suggesting the involvement of other factors in the disease’s molecular mechanism. The role of epigenetic modifications, which was already hypothesized for other diseases (i.e., endometriosis), the etiology of which is associated with the same *HOXA* and *WNT* genes, is possible [23].

Most women with MRKHS have normal ovarian function; however, cases of primary ovarian failure due to ovarian agenesis or dysgenesis have also been reported [8,20].

## 4. 46,XY Phenotypical Females with CAUV

### 4.1. Complete Androgen Insensitivity Syndrome

The complete androgen insensitivity syndrome (CAIS) (OMIM 300068) is an X-linked recessive 46,XY DSD. It is the most common cause of DSD in completely feminized 46,XY patients. The patients have normal female external genitalia and present with primary amenorrhea after normal breast development at puberty. The pubic and axillary hair is absent or scanty, the vagina is short and blind-ending, and the uterus is absent. Discordance between the result of prenatal karyotyping and phenotype of external genitalia at birth as well as the presence of a palpable mass that corresponds to testis in the labia majora or inguinal canal can prompt the diagnosis earlier. About 80% to 100% of CAIS are caused by loss-of-function mutations in the coding region of the androgen receptor (AR) gene (*AR* gene), located on the X chromosome (Xq12), and is composed of 8 exons [41]. The AR is a 920 amino acids–long protein and contains a DNA binding domain (DBD), linked by the short hinge region (HR) to the C-terminal ligand-binding domain (LBD), and the N-terminal domain (NTD) [42]. A complete loss of function mutations, clinically associated with CAIS, occur predominantly in the LBD of the AR protein (encoded by exons 4–8) followed by the NTD of the AR protein (encoded by exon 1) and are usually single base–pair missense substitutions [43,44]. More than 350 loss-of-function mutations of the *AR* gene have been reported in patients with CAIS [42,43]. Nevertheless, in up to 20% of patients with the clinical diagnosis of CAIS and ruled out mutations in the steroid 5α-reductase type 2 gene (*SRD5A2* gene), the molecular cause remains unknown [45]. Individuals with CAIS without mutations in the *AR* gene but disrupted androgen signaling in the genital skin fibroblasts (GFs) were reported to have mutations in the regulatory regions of the *AR* gene (c. -547C>T affecting upstream open reading frame (uORF) in the 5′untranslated region (5′-UTR) of the *AR* gene, generating repression of the AR protein translation) [46]. In another patient with CAIS, with no mutations in the *AR* coding region and undetectable AR protein in the fibroblast from the labia majora, a causative deep intronic mutation c.2450-118A>G in the intron 6 of the *AR* gene was identified [47]. These two discoveries reinforce a consideration that mutations in the AR coregulators and interacting proteins could account for CAIS in patients lacking mutations in the coding region of the *AR* gene [43].

### 4.2. 5α-Reductase Type 2 Deficiency

Steroid 5α-reductase type 2 (SRD5A2) catalyzes the nicotinamide adenine dinucleotide phosphate (NADPH)-dependent reduction of testosterone (T) into dihydrotestosterone (DHT) [48]. The function of the DHT-AR complex is to induce the differentiation of the male urethra, prostate gland, penis, and scrotum during embryogenesis and to induce virilization during puberty. SRD5A2 deficiency (OMIM 264600) is a rare autosomal recessive 46,XY DSD. The *SRD5A2* gene is located at 2p23 and is composed of 5 exons. More than 100 *SRD5A2* gene mutations have been reported in different ethnic groups [48]. Homozygous mutations predominate over compound heterozygous, mostly affecting exons 1 and 4 [49]. Mutations associated with SRD5A2 deficiency were demonstrated to reduce the enzyme’s catalytic efficiency and binding of T or NADPH, yet reliable information on the kinetic consequences of these mutations is scarce [48]. The SRD5A2 deficiency is characterized by high phenotypical variability of the external genitalia differentiation: from hypospadias in men to normal females with subsequent virilization during puberty. It was observed that carriers of the most cited mutations that diminish testosterone affinity (with the most common homozygous p.G34R) had a predominantly female phenotype, while the patients with the most common mutations decreasing enzymatic activity (the most frequent is homozygous p.R227Q) had less severe phenotypes [50]. Nevertheless, a genotype–phenotype correlation is poor, and even in patients carrying the same homozygous or compound heterozygous mutations, variable phenotypes were observed [49]. In a 46,XY patient with female external genitalia at birth and virilization at puberty, the combination of primary amenorrhea, lack of breast development, clitoromegaly, normal pubic hair development, CAUV, and high T level prompt the diagnosis of SRD5A2 deficiency [51]. In patients with a predominantly female phenotype, early medical referral and diagnosis are usually prompted by either clitoromegaly or the presence of palpable masses in the labial folds or the inguinal canals [52]. Low serum concentration of DHT and increased T to DHT ratio of >20 as biochemical criteria of the diagnosis are not always evident [51]. Molecular evaluation (DNA sequencing of the entire *SRD5A2* gene) is necessary for the definitive diagnosis, especially in the pre-pubertal period, when the clinical picture of SRD5A2 deficiency might be very similar to AIS [53]. Nevertheless, in a study of 23 adult 46,XY women with the clinical diagnosis of SRD5A2 deficiency based on urinary steroid profile analysis, sequencing of the SRD5A2 gene revealed pathological mutations and confirmed the diagnosis in 43% (10/23) of subjects [54]. In populations with a high rate of inbreeding, the diagnosis of SRD5A2 deficiency as a cause of 46,XY DSD in a female with an absent uterus might be more probable than CAIS diagnosis [55].

### 4.3. 17β-Hydroxysteroid Dehydrogenase Type 3 Deficiency

17β-hydroxysteroid dehydrogenase type 3 (17β-HSD-3, HSD17B3) is a 310 amino acid–long enzyme encoded by the *HSD17B3* gene located at 9q22.32. HSD17B3 is present almost exclusively in the testicular Leydig cells, and in the presence of cofactor NADPH converts Δ4-androstendione (Δ4-A) to T [56]. HSD17B3 deficiency (OMIM 264300) is a very rare autosomal recessive 46,XY DSD, caused by homozygous or compound heterozygous mutations in the *HSD17B3* gene. Approximately 70 different mutations in this gene have been reported [57]. The most frequent site of identified mutations is in exon 9, followed by the R80 in exon 3 [58]. Ethnic differences were noticed in the preponderance of different mutations, with a predominance of c.277 + 4C>7A in Caucasians and p. R80Q in West Asians [59]. The diagnosis of HSD17B3 deficiency should be considered in patients with primary amenorrhea and a mild degree of virilization. The most frequent presentation of the HSD17B3 deficiency, which might be clinically indistinguishable from AIS or SRD5A2 deficiency, is a 46,XY individual with female external genitalia, hypoplastic vagina, and an absent uterus, with or without mild clitoromegaly, in childhood seeking medical attention because of inguinal or intralabial masses, while at puberty or in adulthood because of virilization or primary amenorrhea [56,59]. The biochemical hallmark of HSD17B3 deficiency is a low T/Δ4-A ratio of <0.8, stimulated by human chorionic gonadotropin (hCG) or baseline in post-pubertal patients (sensitivity 100%, specificity 91%) [60]. Definitive diagnosis requires confirmation by genetic testing.

### 4.4. Leydig Cells Hypoplasia

The Leydig cells hypoplasia (LCH) is a very rare autosomal recessive 46,XY DSD. LCH is due to homozygous or compound heterozygous inactivating or loss-of-function mutations in the luteinizing hormone (LH)/chorionic gonadotropin (CG) receptor gene (*LHCGR* gene) [61]. The *LHCGR* gene is located at 2p16.3 and contains 11 coding exons [62]. The LHCGR is a member of the G protein-coupled receptor superfamily. The mutant LHCG receptors’ impaired function is mainly due to severe receptor intracellular retention and reduced expression at the cell surface [63]. Abnormal development of anatomical sex in 46,XY patients with this disorder results from impaired or completely abolished Leydig cells development and function. Androgen production by testis is partially or fully deficient, due to the inability of Leydig cells to respond to hHG and subsequently, to LH. Sertoli cells are not affected, and therefore AMH secretion during embryogenesis is normal. The predominance of female external genitalia with a blind-ending vaginal dimple and an absent uterus is the severe form of LCH type I (OMIM 238320). The patients have inguinal or intra-abdominal testes and present with a lack of breast development and primary amenorrhea at puberty. The hormonal profile of the patients shows low serum T level (basal and stimulated by hCG), normal male range serum AMH level, elevated serum LH and FSH levels, low serum DHT level, normal serum Δ4-A level. In patients with a severe phenotype, nonsense and missense mutations located in the exons of the *LHCGR* gene, mostly exon 11, were reported [64]. LCH type I is a very rare disorder. In a study that involved a large international cohort of 278 patients with 46,XY DSD, including 37 patients with disorders in androgen synthesis and action, only one patient was diagnosed with LCH [65]. The prevalence is higher in populations with a high degree of parental consanguinity or endogamy, especially in the Arab countries of North Africa and the Middle East [62].

### 4.5. Obstructive Uterovaginal Anomalies

Obstructive anomalies of the reproductive tract that preclude the occurrence of menses involve cervical aplasia, partial vaginal aplasia, complete transverse vaginal septa, and imperforate hymen (respectively, classes C4, V4, and V3 of the ESHRE/ESGE classification [1]). Amenorrhea due to these anomalies is rather cryptomenorrhea, as uterine bleeding occurs but vaginal bleeding does not occur because of the completely obstructed outflow. Therefore, the typical presentation of these anomalies is acute or recurrent lower abdominal pain in an amenorrheic girl. The coexistence of partial vaginal aplasia or transverse vaginal septum with primary or secondary ovarian failure might mimic MRKHS, as the patient, prior to surgical correction and pubertal induction, has a short, blind-ending vagina and a hypoplastic uterus. The knowledge about the genetic background of these particular obstructive anomalies is very limited; however, some studies suggest that Müllerian formation anomalies, including MRKHS, and Müllerian fusion anomalies may have a common etiology [66]. Cases of familial occurrence of an imperforate hymen, both in siblings and across consecutive generations, strongly suggest a genetic background [67].

## 5. Conclusions

In adolescents with syndromic or non-syndromic skeletal or renal malformations, Müllerian aplasia as a cause of primary amenorrhea is highly probable. MRKHS is a complex and heterogeneous disease, usually sporadic. The molecular etiology in the majority of cases remains unknown; however, the contributions of certain causative gene variants were found. In about 10% of MRKHS patients, potentially pathogenic CNVs are identified by array CGH. The *WNT4* gene mutations were proved to cause MRKHS in some patients with hyperandrogenism. CAIS is considered the most common diagnosis in 46,XY females with an absent uterus; however, in populations with a high rate of inbreeding, the probability of a diagnosis of SRD5A2 deficiency might be similar. The offspring of consanguineous parents are also at higher risk of HSD17B3 deficiency and LCH type 1. In contrast to patients with MRKHS, the molecular diagnosis can be established in the majority of patients with these 46,XY DSDs.

## Figures and Tables

**Table 1:** Spectrum of diseases affecting phenotypical females with primary amenorrhea and uterovaginal aplasia.

Disease	OMIM	Karyotype	Genetic Etiology	Gonads
MRKHS type 1	277000	46,XX	Largely unknown	Normal ovaries
MRKHS type 2 (including MURCS association)	601076	46,XX	Largely unknown	Normal ovaries (rarely ovarian agenesis/dysgenesis)
MRKHS and hyperandrogenism	158330	46,XX	*WNT4* mutations	Normal ovaries, hyperandrogenism
CAIS	300068	46,XY	*AR* mutations	Testes, high T
5α-reductase type 2 deficiency	607306	46,XY	*SRD5A2* mutations	Testes, high T
17β-hydroxysteroid dehydrogenase type 3 deficiency	264300	46,XY	*HSD17B3* mutations	Testes, low T
Leydig cells hypoplasia type 1	238320	46,XY	*LHCGR* mutations	Testes, low T

MRKHS—Mayer-Rokitansky-Küster-Hause syndrome, MURCS association—müllerian aplasia, renal aplasia, cervicothoracic somite anomalies association, CAIS—complete androgen insensitivity syndrome, T—testosterone.

## Data Availability

Not applicable.
